# JAG1 overexpression contributes to Notch1 signaling and the migration of HTLV-1-transformed ATL cells

**DOI:** 10.1186/s13045-018-0665-6

**Published:** 2018-09-19

**Authors:** Marcia Bellon, Ramona Moles, Hassiba Chaib-Mezrag, Joanna Pancewicz, Christophe Nicot

**Affiliations:** Department of Pathology and Laboratory Medicine, Center for Viral Pathogenesis, University of Kansas Medical Center, 3901 Rainbow Boulevard, MS 3046, Kansas City, KS 66160 USA

**Keywords:** HTLV-I, ATL, Notch, JAG1, NF-κB, miR-124, STAT3, NFATc1

## Abstract

**Background:**

HTLV-1 is a retrovirus that infects over 20 million people worldwide and is responsible for the hematopoietic malignancy adult T cell leukemia (ATL). We previously demonstrated that Notch is constitutively activated in ATL cells. Activating genetic mutations were found in Notch; however, Notch signaling was also activated in the absence of genetic mutations suggesting the existence of other mechanisms.

**Methods:**

We analyzed the expression of Notch receptor ligands in HTLV-I-transformed cells, ATL patient-derived cell lines, and fresh uncultured ATL samples by RT-PCR, FACS, and immunohistochemistry. We then investigated viral and cellular molecular mechanisms regulating expression of JAG1. Finally, using shRNA knock-down and neutralizing antibodies, we investigated the function of JAG1 in ATL cells.

**Results:**

Here, we report the overexpression of the Notch ligand, JAG1, in freshly uncultured ATL patient samples compared to normal PBMCs. We found that in ATL cells, JAG1 overexpression relies upon the viral protein Tax and cellular miR-124a, STAT3, and NFATc1. Interestingly, our data show that blockade of JAG1 signaling dampens Notch1 downstream signaling and limits cell migration of transformed ATL cells.

**Conclusions:**

Our results suggest that targeting JAG1 can block Notch1 activation in HTLV-I-transformed cells and represents a new target for immunotherapy in ATL patients.

## Background

The Notch pathway is one of the most frequently activated signaling pathways in human malignancies. Activating mutations or amplification of the Notch pathway is commonly reported in various types of human cancer. T cell and glial cell cancers are especially prone to having an oncogenic Notch pathway, since Notch plays a key role in differentiation and development in these cell types [1]. Activated Notch1 has also been shown to play important roles in virus-associated cancers such as Kaposi’s sarcoma (KSHV) [2] and HCV- or EBV-associated lymphoma [3, 4]. The human Notch family includes four receptors, Notch 1–4, and five ligands, delta-like ligand 1 (DLL1), delta-like ligand 3 (DLL3), delta-like ligand 4 (DLL4), Jagged-1 (JAG1), and Jagged-2 (JAG2) [5]. In physiological conditions, interactions between these ligands and the extracellular domain of the Notch receptor, which is located on the cellular surface of neighboring cells, lead to the proteolytic cleavage and release of the Notch intracellular domain (NICD). NICD then translocates to the nucleus where it interacts with DNA-binding proteins and activates target genes. Termination of Notch1 signaling can occur at, or downstream of, the Notch receptor through ubiquitin ligases Itch/AIP4 (itchy E3 ubiquitin protein ligase) or Nedd4 (neural precursor cell-expressed developmentally downregulated protein 4) [6, 7]. NICD can also be phosphorylated by glycogen synthase kinase 3 beta (GSK3β), which regulates its interaction with the E3 ubiquitin ligase, FBXW7 (F-box and WD repeat domain containing 7). This promotes ubiquitination and proteasome-mediated degradation of NICD [8].

The average life expectancy for HTLV-I-associated acute adult T cell leukemia (ATL) is less than 12 months, and since there is no cure for the disease and treatment options are very limited, new therapeutic targets are greatly needed [9]. Although the etiologic agent has been well characterized, a mechanistic understanding of the initiation and progression of this disease has been elusive. The low incidence and long latency of HTLV-I-associated ATL suggest that in addition to viral infection, accumulation of genetic mutations and genomic alterations is required for cellular transformation [10]. In the early stages of the transformation process, the viral transcriptional activator protein Tax plays an essential role by disrupting the normal state of many cellular signaling pathways, inactivating tumor suppressors, increasing the mutation rate, and inhibiting DNA repair pathways [11, 12]. We have previously demonstrated that HTLV-I-transformed cells and ATL cells display constitutive activation of Notch1 signaling [13]. We further demonstrated that inhibition of Notch1 signaling by a gamma-secretase inhibitor (GSI) reduced ATL tumor cell proliferation and tumor formation in a xenograft mouse model of ATL [13].

Notch1 activating mutations have been reported in various cancers [14]. Genetic aberrations in hematological malignancies frequently involve Notch receptors or its regulators, such as FBXW7. In T cell acute lymphocytic leukemia (T-ALL) patients, these mutations usually cluster at the hetero-dimerization (HD) and proline-glutamate-serine-threonine-rich (PEST) domains of Notch [15]. HD domain mutations are characterized by ligand-independent constitutive cleavage of the Notch1 receptor, resulting in increased expression of NICD. In contrast, mutations in ATL patients do not occur in the HD domain, but instead occur solely in the PEST domain of NICD. Mutations in the PEST domain have been shown to increase the stability of NICD. As much as 30% of ATL patients harbor mutations within the PEST domain of NICD, thereby preventing proper ubiquitination and NICD proteasome degradation [13]. These observations and the lack of mutations within the HD domain of NICD in ATL patients suggests that interaction between the Notch receptor and one of its ligands is required for activation of Notch signaling in ATL cells.

To better understand the regulation of the Notch signaling pathway in ATL, we investigated the expression of Notch receptor ligands. Here, we show that HTLV-I-transformed and fresh uncultured ATL cells overexpress JAG1 and to a lesser extent DLL4. We further demonstrate that the viral Tax protein, but not HBZ, stimulates expression of JAG1 in part through activation of the nuclear factor kappa B (NF-κB) pathway. In ATL cells, the expression of JAG1 is also correlated with the transcriptional regulators, STAT3 (signal transducer and activator of transcription 3) and NFATc1 (nuclear factor of activated T cells 1). We also show that miR-124 expression can target STAT3 and NFATc1 to lower JAG1 expression in ATL cells. Finally, we found that blockade of JAG1 signaling by shRNA or neutralizing antibodies dampened Notch signaling and limited cell migration of transformed ATL cells. Our results suggest that JAG1 may represent a new target for immunotherapy in ATL patients.

## Methods

### Cell cultures and ATL patient samples

The HTLV-I-transformed (IL-2 independent) cell lines (MT2, MT4, C8166, and C91PL), ATL-like cell lines (ATLT, ATL25, ED-40515(−), and TL-Om1), and ALL cell lines (Jurkat and Molt4) were grown in RPMI 1640 with 10% fetal bovine serum. The HTLV-I-immortalized (IL-2 dependent) cell lines (LAF and 1185) and the ATL-like cell lines (ATL43T, ATL55T, KOB, KK1, SO4, and LM-Y1) were grown in media with 20% serum and 50 U/mL IL-2. The ATL patient samples used in this study were previously described in another publication [13, 16]. Samples were obtained after informed consent after internal institutional review board approval, respecting the regulations for the protection of human subjects. The present study control samples consist of isolated peripheral blood mononuclear cells (PBMCs) from healthy HTLV-1-negative donors that have been previously reported [16]. Pharmacological inhibitors used to treat cells include STAT3 inhibitor, S3I-201 (Calbiochem), and NFAT inhibitor (Cayman Chemical Company).

### RNA extraction and RT-PCR

RNA was extracted using TRIzol (Invitrogen) followed by DNase I treatment and reverse transcription with an RNA-to-cDNA synthesis kit (Applied Biosystems). The StepOnePlus Real-time PCR System (Applied Biosystems) was used in the study to quantify the expression of the genes of interest. The following primers were used in this study with iTaq Universal SYBR green (Bio-Rad): GAPDH (S-GAAGGTGAAGGTCGGAGTC and AS-GAAGATGGTGATGGGATTTC), STAT3 (S-GATTGACCAGCAGTATAGCCGCTTC and AS-CTGCAGTCTGTAGAAGGCGTG), pre-miR-124a (S-AGGCCTCTCTCTCCGTGTTC and AS-CAGCCCCATTCTTGGCATTC), JAG1 (S-ATCGTGCTGCCTTTCAGTTT and AS-GATCATGCCCGAGTGAGAA), JAG2 (S-GTCGTCATCCCCTTCCAGT and AS-CTCCTCATTCGGGGTGGTAT), DLL4 (S-AGGCCTGTTTTGTGACCAAG and AS-GTGCAGGTGTAGCTTCGCT), IL-8 (S-CTGATTTCTGCAGCTCTGTGTG and AS-CAGACAGAGCTCTCTTCCATCAG), RelA (S-CTCTGCTTCCAGGTGACAGT and AS-TCCTCTTTCTGCACCTTGTC), Hes1 (S-CTGGAAATGACAGTGAAGCACCT and AS-ATTGATCTGGGTCATGCAGTTG), Hey1 (S-CCGAGATCCTGCAGATGACC and AS-CCCGAAATCCCAAACTCCGA), and VEGF (S-TCTACCTCCACCATGCCAAGT and AS- GATGATTCTGCCCTCCTCCTT). NFATc1 was detected using iTaq Universal Probes (Bio-Rad) with the following primers: NFATc1 (S-CCATCCTCTCCAACACCAAA, AS-GTCTCTCCTTTCCGAAGTTCAA, and probe-ACTGTGCCGGAATCCTGAAACTCA).

### Antibody staining and fluorescence-activated cell sorting (FACS)

Cells were collected, washed twice with PBS, and stained with the antibodies according to the manufacturer’s instructions. The samples were then washed twice with PBS before analysis with a LSR II flow cytometer. Fixation with PFA 4% was included for JAG1 antibody staining before incubation with the antibody. The following antibodies were used: FITC anti-human JAG1 (Sino Biological Inc.), FITC Mouse IgG2a isotype control (BD PharMingen), and PE anti-Human DLL4 (Biolegend).

### Immunohistochemistry

Cells were grown on a coverslip slide coated with poly-Lysine or cytospined onto a coverslip. Cells were then fixed with 4% PFA. Immunohistochemistry (IHC) was performed using an EXPOSE HRP/DAB Detection IHC Kit (Abcam), with JAG1 (R&D Systems) and DLL4 (Abcam) antibodies, counterstaining with Mayer’s hematoxylin (Lillie’s modification) and Bluing reagent (ScyTek). Images were taken with a Nikon Eclipse 80i microscope (Nikon Instruments, Inc., Melville, NY) with a × 60 objective lens and a Nikon DSFI1 camera.

### Plasmids

The HTLV-1 Tax gene was cloned into the pTRIPZ, lentiviral inducible vector, engineered to become Tet-On. The pTRIPZ vector contains puromycin resistance, which was used to select a stable cell line. The stable cell lines were incubated in the absence or presence of doxycycline to induce the expression of the viral protein Tax. Tax and HBZ genes were also cloned in the pSIH1-green fluorescent protein (GFP) lentiviral vector. A shRNA against JAG1 was cloned into the pSIH1-GFP vector. The pTRIPZ and miR-124a/pTRIPZ stable lines and the miR-124a/pCDNA construct are previously described [16]. Luciferase assays were performed using the Dual-Luciferase Reporter System (Promega). The wild-type 3′UTR of NFATc1 was cloned into a modified pGL3-Promoter luciferase vector (Promega) with the primers S-GGTCTAGATTGCCACATTGGAGCACTCAGTTCAGC and AS-CCGAATTCCGGCTTTATTGGATCTATTTCCTAACTAC. Mutant NFATC1 3′UTR sites were generated using the site-directed mutagenesis kit (Stratagene).

### Western blotting

Cell lysates were separated on SDS-PAGE gels followed by electroblotting to polyvinylidene difluoride membranes and probed with Actin (sc-1615), NFATC1 (7A6), and a Tax mouse monoclonal antibody from the NIH AIDS Reagent Program, HTLV-I Tax Hybridoma (168B17), and with appropriate secondary antibodies from Santa Cruz Biotechnology.

### XTT proliferation assays

Cell viability and proliferation were measured by Cell Proliferation Kit II (XTT) (Roche) according to the manufacturer’s instructions. One hundred microliters of cells were seeded in a 96-well plate, and 50 μL of XTT labeling mixture was added to each well and incubated for 4–6 h. Spectrophotometry was used to measure the absorbance at 450 and 620 nm. The results were plotted as mean, and the standard deviation is shown from at least two independent experiments.

### Scratch-wound assays

Cells were plated in a 12-well plate, and when the cells reached confluence, they were treated with 3 μg/ml of JAG1-neutralizing antibody. After 3 days, the media was removed and replaced with fresh media with 3 μg/ml of JAG1-neutralizing antibody. After 3 days, the p1000 tip was used to scratch the plate. The plate was then washed twice with PBS, and fresh media with neutralizing antibody (3 μg/ml) was added. After 48 h, the cells were fixed with cold methanol (MeOH) and then stained with crystal violet dye (0.5% MeOH) for 20 min at room temperature. Images were taken with an Olympus 1x71 Inverted Microscope with a × 40 objective lens.

### Statistical analysis

Experiments in figures were performed multiple times in duplicate. Representative results were shown in the final figures. *P* values were calculated by using paired and two-tailed Student’s *t* test. In the figures, asterisk indicates *p* value < .05, two asterisks indicate *p* value < .01, and three asterisks indicate *p* value < .001. Correlation analysis was performed by using Pearson’s correlation. The Pearson’s correlation coefficient, coefficient of determination, and *p* values are reported in the figures.

## Results

### Overexpression of JAG1 in HTLV-I-transformed and ATL-derived patient cell lines

**Fig. 1:**
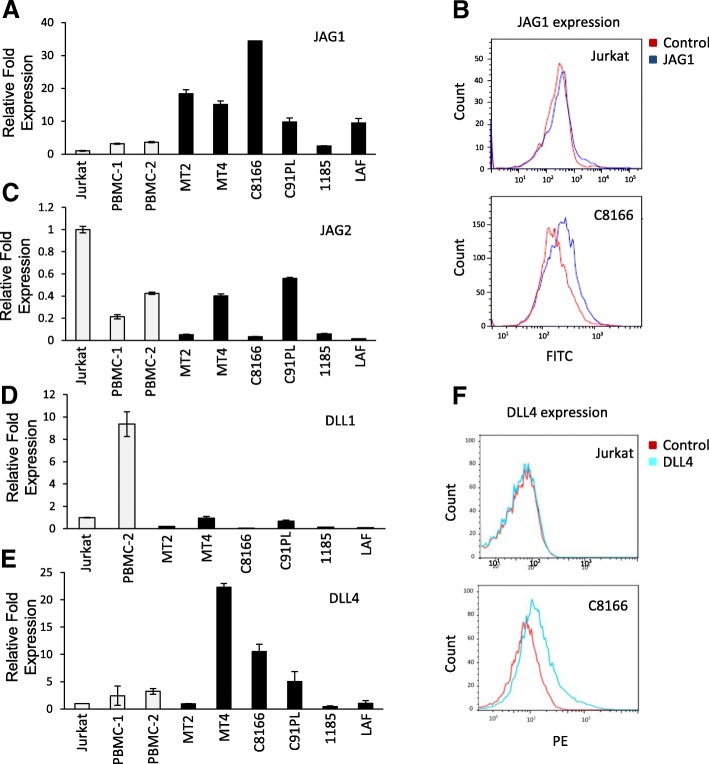
Expression of Notch ligands in HTLV-I cell lines. **a** Real-time PCR was performed on JAG1 from cDNA derived from HTLV-I-immortalized and transformed cells (MT2, MT4, C8166, C91PL, 1185, and LAF). The non-HTLV-I Jurkat T cell line and normal PBMCs isolated from HTLV-1-negative donors were used as controls. Real-time PCR was performed in duplicate, and samples were normalized to GAPDH expression. Fold change was calculated by comparing values with Jurkat normalized JAG1 expression. **b** Antibody staining of JAG1 surface expression was performed on the HTLV-I-transformed cell line C8166 and negative control Jurkat cells. Cells stained with FITC Mouse IgG2a isotype were used as a control. Red peaks indicate the isotype control, while blue peaks indicate the JAG1 antibody. Bar diagrams representing the FACS results are provided. **c** Same as **a** for JAG2 (**d**). Same as **a** for DLL1. **e** Same as **a** for DLL4. **f** Antibody staining for cell surface expression of DLL4 was performed on the HTLV-1-transformed cell line C8166 and negative control Jurkat with an antibody against DLL4. Unstained cells were used as a control. Red peaks indicate the control, while blue peaks indicate the DLL4 antibody

**Fig. 2:**
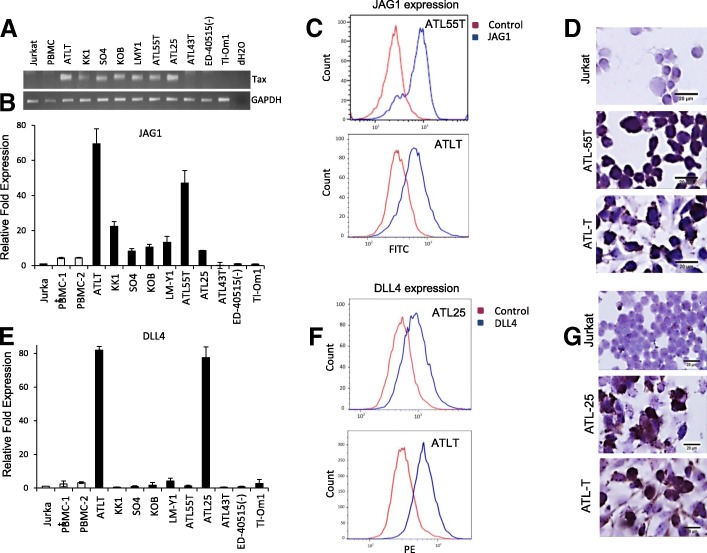
Expression of notch ligands in ATL cell lines. **a** PCR on Tax and GADPH expression from cDNA derived from ATL-derived cell lines and negative controls, Jurkat and PBMCs. GAPDH expression was used as an internal control. Real-time PCR was performed on JAG1 (**b**) or DLL4 (**e**) using cDNA from ATL-derived cell lines (ATLT, KK1, SO4, KOB, LM-Y1, ATL55T, ATL-5, ATL43T, ED-40515(−), and Tl-Om1). HTLV-I-negative Jurkat T cell line and normal PBMCs isolated from HTLV-1-negative donors were used as controls. Real-time PCR was performed in duplicate, and samples were normalized to GAPDH expression. Fold change was calculated by comparing values with Jurkat normalized JAG1 expression. Antibody staining of JAG1 (**c**) or DLL4 (**f**) was performed on the ATL cell lines, ATL55T and ATLT, and Jurkat (data included in Fig. 1). Cells were stained with the antibody against JAG1 and analyzed via a flow cytometer. FITC Mouse IgG2a isotype was used as an internal control. Red peaks indicate the control, while blue peaks indicate JAG1 or DLL4 antibodies. Bar diagrams representing the FACS results are provided. Immunohistochemistry of JAG1 on ATL55T and ATLT (**d**) or DLL4 on ATL25 and ATLT (**g**) and Jurkat as a negative control was performed. Images were taken with a × 60 objective lens

We used RT-PCR to test the expression of Notch receptor ligands JAG1, JAG2, DLL1, and DLL4 in HTLV-I-infected immortalized (IL-2-dependent) and transformed (IL-2-independent) cell lines compared to the HTLV-I-uninfected T cell line, Jurkat, and PBMCs isolated from healthy donors. Generally, Notch receptor ligands JAG2 and DLL1 were downregulated when compared to normal PBMCs (Fig. 1c, d). Overexpression of the Notch receptor ligand JAG1 was detected in five of six HTLV-I cell lines tested when compared to HTLV-I-negative cells. Only HTLV-I-immortalized 1185 cells did not significantly overexpress JAG1 (Fig. 1a). To confirm that the JAG1 ligand was overexpressed on the cell surface of HTLV-I-infected cells, we used JAG1 antibody staining followed by FACS analysis. Our analysis confirmed high cell surface expression of JAG1 (Fig. 1b), suggesting that it may play a role in the constitutive activation of Notch signaling in HTLV-I-infected cells. Finally, expression of the Notch receptor ligand DLL4 was variable in HTLV-I-infected cell lines compared to HTLV-I-negative cells, but was overexpressed on the cell surface of MT4 and C8166 transformed cells (Fig. 1e, f). We next investigated the expression of Notch receptor ligands JAG1 and DLL4 in a series of ATL patient-derived cell lines. These cell lines are of ATL origin and display varying levels of the HTLV-I oncoprotein, Tax (Fig. 2a). Overexpression of JAG1 was detected in seven out of ten ATL cell lines tested (Fig. 2b), and cell surface expression was confirmed by FACS and IHC analysis (Fig. 2c, d). In contrast, DLL4 was found to be overexpressed in only two ATL cell lines, ATLT and ATL25 (Fig. 2e). These results were validated by using FACS and IHC, using Jurkat cells as a negative control (Fig. 2f, g).

### Virus-encoded Tax, but not HBZ, activates JAG1 expression through NF-κB

**Fig. 3:**
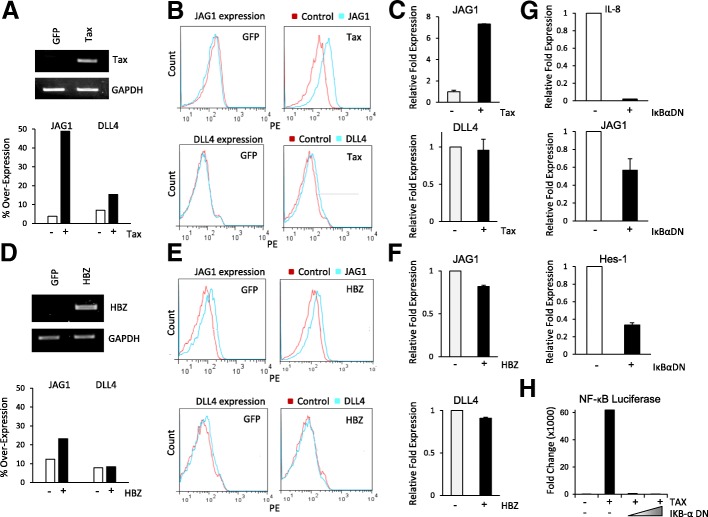
HTLV-I, Tax, induces expression of JAG1 through NF-κB. **a** PCR was performed to detect Tax expression on cDNA derived from the ALL cell line, Molt4, infected with the lentivirus pSIH1-Tax. Cells infected with the empty vector pSIH1. **b** Antibody staining of JAG1 and DLL4 was performed on Molt4 cells infected with pSIH1-Tax and pSIH1-GFP. Red peaks indicate the control, while blue peaks indicate the JAG1 or DLL4 antibodies. Bar diagrams representing the FACS results are provided. **c** Real-time PCR of JAG1 and DLL4 expression on cDNA derived from Molt4 infected with pSIH1-Tax. Cells infected with the empty vector expressing pSIH1-GFP were used as a control. The expression of JAG1 and DLL4 were normalized to GAPDH expression. Results were plotted as mean ± standard deviation from at least two independent experiments. **d** PCR was performed to detect HBZ expression on cDNA derived from Molt4 infected with a lentivirus pSIH1-HBZ. Cells infected with the empty vector expressing pSIH1-GFP were used as a control. GAPDH expression was used as an internal control. **e** Antibody staining of JAG1 and DLL4 was performed on the Molt4 cells infected pSIH1-HBZ and pSIH1-GFP. Red peaks indicate the control, while blue peaks indicate the JAG1 or DLL4 antibodies. Bar diagrams representing the FACS results are provided. **f** Real-time PCR to detect JAG1 and DLL4 expression on cDNA extracted from Molt4 cells infected with pSIH1-HBZ. Cells infected with the empty vector expressing pSIH1-GFP were used as a control. Real-time PCR was performed in duplicate, and samples were normalized to GAPDH expression. Results were plotted as mean ± standard deviation from at least two independent experiments. **g** Real-time PCR analysis of IL-8, JAG1, and Hes-1 expression on cDNA from Molt4 cells infected with pSIHI-GFP and a lentivirus vector expressing IκB-α-DN mutant. Cells infected with the empty vector expressing pSIH1-GFP were used as a control. Extracts were analyzed 48 h after infection and normalized to GAPDH expression. Real-time PCR was performed in duplicate. Results were plotted as mean ± standard deviation from two independent experiments. **h** NF-κB luciferase was performed on 293T cells transfected with Tax and/or IκBα-DN plasmids

We next investigated the molecular mechanism associated with increased JAG1 expression in HTLV-I-transformed cell lines. The viral Tax gene encodes for a 40-kDa nuclear phosphoprotein that has pleiotropic effects on HTLV-I-infected cells [17]. Tax has been shown to positively regulate the transcription of many cellular genes. Furthermore, JAG1 expression was elevated in all ATL lines that had detectable Tax expression (Fig. 2a). JAG1 expression was lower in ED-40515(−), ATL43T, and Tl-Om1, where Tax expression from cDNA was undetectable by PCR analysis. To test whether Tax can activate JAG1 expression, we transiently expressed Tax in Molt4, an HTLV-I-negative T cell line, where Tax is not expressed. Successful delivery and expression of Tax by lentiviral transduction was confirmed by RT-PCR (Fig. 3a). Expression of JAG1 following transduction of Tax was investigated by FACS and RT-PCR. Results from these studies confirmed a strong induction of JAG1 mRNA and cell surface expression in Tax-transduced cells (Fig. 3b, c). In contrast, parallel analyses on the same transduced cells indicated that Tax had no significant effects on DLL4 expression (Fig. 3b, c). These results were also independently confirmed using an inducible Jurkat Tax Tet-On cell line (data not shown). Since HTLV-I Tax is a potent inducer of the NF-κB pathway, we investigated if Tax-mediated JAG1 overexpression occurred in an NF-κB-dependent manner. To this end, we constructed and transduced Tax-expressing MT4 cells with an IκBα (NF-κB inhibitor alpha) dominant negative mutant lentivirus able to suppress Tax-mediated NF-κB canonical activation [18]. As expected, transduction with IκBα-DN resulted in significant suppression of interleukin-8 (IL-8) mRNA expression (Fig. 3g), a well-known NF-κB target gene [19]. Consistent with a role for Tax-mediated NF-κB activation in controlling JAG1 expression, JAG1 mRNA expression was also downregulated following transduction of the IκBα dominant negative mutant (Fig. 3g). Notably, the Notch1 target gene, hairy and enhancer of split 1 (Hes1), was also significantly suppressed (Fig. 3g), suggesting that Tax-mediated JAG1 expression partly contributes to Notch signaling. In order to confirm that the IκBα-DN plasmid used could indeed block Tax transactivation of NF-κB, we transfected cells with NF-κB luciferase (Fig. 3h). Tax expression increased NF-κB luciferase, but was blocked in the presence of increasing IκBα-DN plasmid. The HTLV-I HBZ gene is reportedly expressed at the mRNA level in most ATL cells, although its expression seems quite variable among samples and laboratories [20]. We tested if HBZ might be involved in the activation of JAG1 or DLL4 expression in ATL cells. Transduction of Molt4 cells with an HBZ lentiviral vector was performed, and both JAG1 and DLL4 expression were analyzed by RT-PCR and immunostaining. Our data suggests that HBZ does not play any significant role in the expression of JAG1 and/or DLL4 in ATL cells (Fig. 3d–f).

### Regulation of JAG1 expression through direct and indirect mechanisms involving miR-124a, STAT3, and NFATc1 in ATL cells

**Fig. 4:**
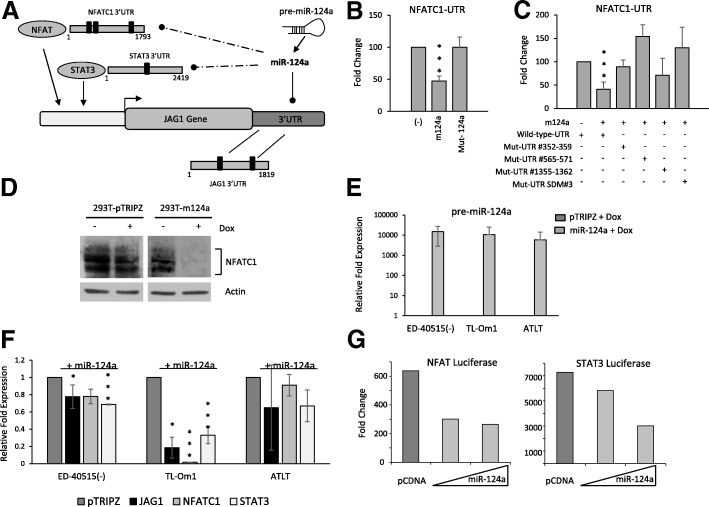
miR-124a inhibits JAG1 expression directly and indirectly through STAT3 and NFATc1. **a** Schematic representation of the JAG1 transcript and its interplay with NFAT, STAT3, and miR-124. Solid black marks represent miR-124a binding sites within the 3′UTR of JAG1 (2 sites), STAT3 (1 site), and NFATc1 (3 sites). **b**, **c** pCDNA, miR-124a/pCDNA, or mutant miR-124a/pCDNA (**b**) (mutated miR-124a sequence) were transfected into 293T cells along with wild-type or mutant (**c**) NFATC1-UTR-pGL3 and the RL-TK plasmid. For mutant NFATC1-UTR, mutations were made at single miR-124a binding sites (#352-359, #565-571, or #1355-1362) or at all three mir-124a binding sites (Mut-UTR SDM#3). Forty-eight hours after transfection, cell lysates were measured for firefly (NFATC1 3′UTR) and renilla (RL-TK, internal control) activity. All luciferase was performed at least twice, and standard deviation is shown. Fold change was calculated compared to cells transfected with empty vector. **d** Detection of NFATC1 in stable 293T-pTRIPZ or –miR-124a cells induced 72 h with 2 μg/ml Dox. **e** Pre-miR-124a expression was detected by RT-PCR on ED-40515(−)-, Tl-Om1-, and ATLT-pTRIPZ and miR-124-pTRIPZ Tet-On inducible lines. **f** STAT3, NFATc1, and JAG1 expression were detected by RT-PCR on ED-40515(−)-, Tl-Om1-, and ATLT-pTRIPZ and miR-124-pTRIPZ Tet-On inducible lines. For **e** and **f**, 2 μg/ml Dox was added every day for 72 h. Post-induction, RT-PCR was performed and samples were normalized to GAPDH expression. Results are plotted as the average fold change from pTRIPZ-induced lines from at least two independent experiments. **g** 293T cells were transfected with pCDNA control or miR-124a/pCDNA, along with NFAT and STAT3 reporter luciferase vectors. Results are represented as a fold change compared to pCDNA transfected cells

Since Tax expression is retained in only 30% of ATL cells and HBZ did not play a significant role in activation of JAG1, we next investigated alternative pathways that could explain the high levels of JAG1 in ATL cells [21]. MicroRNAs (miRNAs) are transcriptional and post-transcriptional regulators of gene expression that are involved in many pathological conditions, including cancer [22]. A previous study identified JAG1 as a direct target of miRNA-124a [23]. Interestingly, our laboratory has shown that miR-124a is significantly downregulated in transformed cell lines and in primary ATL patient samples [16]. In addition to its direct effects on JAG1, miR-124a can also target STAT3 and NFATc1, two transcription factors known to regulate JAG1 gene expression (Fig. 4a). We have previously shown that STAT3 is a direct target of miR-124a [16]. In order to demonstrate a role for miR-124a inhibition of NFATc1, we first confirmed that NFATc1 is a direct target of miR-124a by 3′UTR luciferase assays. We cloned the full-length NFATc1 3′UTR into a pGL3-luciferase vector and showed that miR-124a overexpression led to a statistically significant loss in NFATc1 3′UTR activity (Fig. 4b). The NFATc1 3′UTR contains three miR-124a binding sites. Mutation of individual miR-124a binding sites within the NFATc1 3′UTR led to a loss of miR-124a inhibition to varying degrees (Fig. 4c). However, loss of all three miR-124a binding sites led to complete loss of miR-124a inhibition (Fig. 4c). In addition, mutated miR-124a lost the ability to inhibit NFATc1 3′UTR luciferase activity compared to wild-type miR-124a (Fig. 4b). To confirm that miR-124a can target NFATc1 protein levels, we generated miR-124a/pTRIPZ inducible 293T cells. Induction of miR-124a expression led to a loss of NFATc1 protein levels (Fig. 4d), whereas no loss was found in control, pTRIPZ, inducible cells. Since miRNA regulation of a target gene is context-dependent, we next investigated the role of miR-124a in the overexpression of JAG1 in the context of ATL cells. To this end, we used three TET-ON inducible cell lines expressing miR-124a under the control of an inducible promoter. The addition of doxycycline to the culture media efficiently induced expression of miR-124a (Fig. 4e), which was associated with a significant decrease in JAG1 levels of expression, in two out of three ATL lines (Fig. 4f). Expression of miR-124a in ATLT still led to a decrease in JAG1 expression; however, the level was not significant, possibly due to varying degrees of miR-124a expression after doxycycline induction. Finally, we found that miR-124a could not only suppress the expression of the STAT3 gene in ATL cell lines, but could also suppress NFATc1 mRNA (Fig. 4f). Consistent with these observations, miR-124a was able to inhibit both STAT3- and NFAT-dependent signaling as demonstrated by reduced luciferase activity from promoter reporter assays (Fig. 4g).

### Overexpression of JAG1 in vivo in freshly isolated uncultured ATL patient RNA samples

**Fig. 5:**
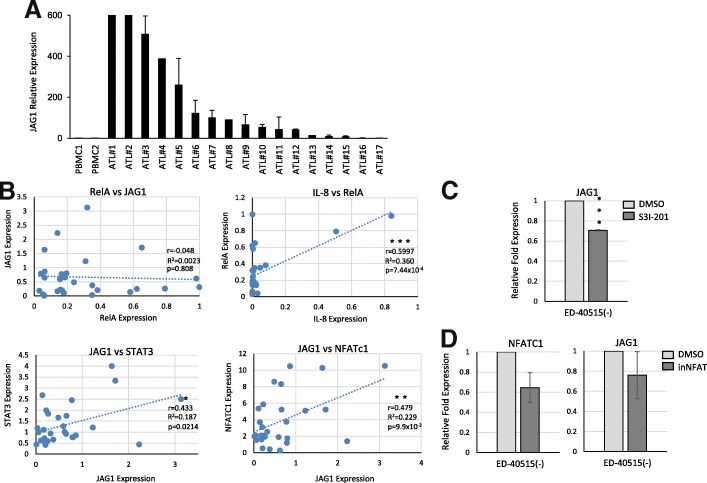
Primary ATL patients overexpress JAG1, which correlates with STAT3 and NFATc1. **a** Real-time PCR analysis of JAG1 expression from cDNA derived from uncultured PBMCs of ATL patients and HTLV-1-negative donors. Samples were normalized to GAPDH expression, and the fold change was calculated by comparing values to healthy donor(s) normalized gene expression. **b** Real-time PCR analysis of JAG1, IL-8, RelA, STAT3, and NFATc1 expression from cDNA derived from PBMCs of patients with ATL and HTLV-1-negative donors. Samples were normalized to GAPDH expression, and the fold change was calculated by comparing values to healthy donor normalized gene expression. Correlation analysis was performed on JAG1 versus RelA, IL-8 versus RelA, JAG1 versus STAT3, and JAG1 versus NFATC1 in ATL patients. The Pearson’s correlation coefficient (*r*), coefficient of determination (*R*^2^), and *p* value of the Pearson’s correlation coefficient are reported. **c**, **d** ED-40515(−) cells were treated 24 h with 50 μM S3I-201 (**c**) or 48 h with 100 μM NFAT inhibitor (inNFAT) (**d**). RT-PCR was performed on JAG1 expression, normalized to GAPDH control. For inNFAT, expression of NFATC1 was also noted. Experiments were performed at least twice, and fold change was calculated from the average repression compared to DMSO control samples

We next investigated the expression of JAG1 mRNA in freshly isolated uncultured PBMCs from acute ATL patients. Our analysis shows that DLL4 was not consistently overexpressed in patient samples (data not shown). In contrast, JAG1 was significantly overexpressed in 12/17 ATL samples (70.6%) when compared to a healthy donor PBMC (Fig. 5a). Since Tax expression is variable to undetectable in ATL patients and NF-κB can induce JAG1 expression, we then tested whether the elevated JAG1 levels in ATL patients were due to an activated NF-κB pathway. Expression of the p65 subunit of NF-κB, RelA, is one of several markers used to test for NF-κB activity. The analysis of RT-PCR expression data for RelA and JAG1 showed no correlation between RelA expression and JAG1 in ATL patients (Fig. 5b). This was in contrast to IL-8, a known downstream target of NF-κB, which strongly correlated with RelA expression in ATL patient samples (*r* = 0.5997). This indicates that NF-κB regulation of JAG1 in HTLV lines occurs through Tax expression and suggests that a Tax-independent mechanism elevates JAG1 RNA in ATL patients. Our previous work demonstrated that over half of ATL patients have high gene expression of STAT3 [16]. We found that NFATc1 gene expression was also high in a majority of ATL patients (data not shown). We then tested the expression of STAT3 and NFATc1 by RT-PCR and found both genes were positively correlated with JAG1 expression in ATL patients (STAT3, *r* = 0.433, and NFATc1, *r* = 0.479) (Fig. 5b). This suggests that STAT3 and NFATc1, which are at least partially regulated by miR-124a, could transcriptionally upregulate JAG1 expression in ATL patients. To further test this hypothesis, we treated an ATL line (ED-40515(−)) with specific pharmacological inhibitors to STAT3 (S3I-201) and NFAT (NFAT inhibitor, inNFAT). RT-PCR confirmed that inhibition of either the STAT3 or the NFAT pathway leads to loss of JAG1 expression in ATL lines; however, the result was only statistically significant for S3I-201 (Fig. 5c, d). The inNFAT prevents dephosphorylation of calcineurin-mediated dephosphorylation of NFAT, thereby preventing calcineurin binding. To test if the inhibitor was working, we performed RT-PCR on NFATc1 levels, since NFATc1 can auto-amplify its own transcription [24]. We found only a 40% loss in NFATc1 transcription following the addition of inNFAT to ED-40515(−) (Fig. 5d). It is possible that higher concentrations of drug are needed in order to fully downregulate the NFAT pathway in ATL cells.

### Inhibition of JAG1 signaling dampened Notch1 signaling and migration of ATL cells

**Fig. 6:**
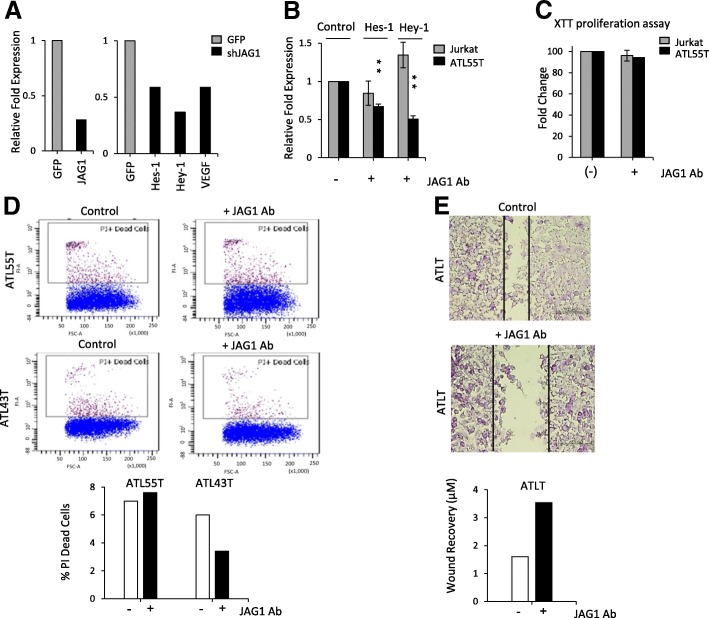
Repression of JAG1 alters Notch signaling and wound healing in ATL lines. **a** RT-PCR was performed on JAG1 from cDNA derived from ATL cell line ATL55T infected with a lentivirus pSIH1-shRNA against JAG1. Cells infected with the empty vector expressing pSIH1-GFP were used as a control. The expression of JAG1 was normalized to GAPDH expression. RT-PCR was then performed on Hes-1, Hey-1, and VEGF from cDNA derived from ATL55T cells infected with pSIH1-shRNA against JAG1. Cells infected with the empty vector expressing pSIH1-GFP were used as a control. Real-time PCR was performed in duplicate, and samples were normalized to GAPDH expression. **b** Real-time PCR was performed on Hes-1 and Hey-1 on cDNA extracted from ATL55T and Jurkat cells incubated for 6 days with neutralizing antibody against JAG1 (3 μg/ml). Real-time PCR was performed in duplicate, and samples were normalized to GAPDH expression. **c** Cell proliferation was measured using XTT assay in ATL55T and Jurkat cells incubated for 6 days with neutralizing antibody against JAG1 (3 μg/ml). Results were plotted as mean ± standard deviation from at least two independent experiments. **d** PI staining was performed to study cell death in ATL55T and ATL43T cells incubated for 6 days with neutralizing antibody against JAG1 (3 μg/ml). Media with antibody was replaced every 3 days, and fresh antibody was added (3 μg/ml). Cells were analyzed for apoptosis by FACS analysis. Bar diagrams representing the FACS results are provided. **e** Wound healing was performed on confluent ATLT cells treated with 3 μg/ml of JAG1-neutralizing antibody for 72 h. After 72 h, the p1000 tip was used to scratch the plate. The plate was then washed, and fresh media with neutralizing antibody was added. After 48 h, the cells were fixed and images were taken with a × 40 objective lens. Bar diagrams representing the wound healing results are provided

In order to validate the significance of JAG1 overexpression in the activation of Notch1 signaling in HTLV-I-transformed cells, we interrupted JAG1 signaling in ATL cells. Efficient downregulation of JAG1 was obtained by lentiviral delivery of JAG1 shRNA into the ATL line, ATL55T (Fig. 6a). Suppression of JAG1 expression was associated with downregulation of the Notch target genes VEGF (vascular endothelial growth factor), Hes1, and Hey1 (hairy-related transcription factor 1) (Fig. 6a). These results confirm that in ATL cells, JAG1 signaling is involved in the activation of the Notch1 pathway. To confirm these results, we then exposed HTLV-I-transformed cells to a JAG1-neutralizing antibody, which can selectively block the binding between the ligand and its receptor. In this assay, we used Jurkat and ATL55T, which display low and high levels of JAG1 expression, respectively. Exposure of Jurkat cells to a JAG1-neutralizing antibody had no significant effect on the expression of the Notch target genes, Hes1 and Hey1 (Fig. 6b). In contrast, blockade of JAG1 by neutralizing antibody resulted in strong inhibition of Hes1 and Hey1 expression in ATL55T (Fig. 6b). Together, these results confirmed that JAG1 overexpression is partly involved in the activation of Notch signaling in ATL cells and may represent a novel therapeutic target. To test the biological significance of JAG1 in ATL cells, we measured cellular proliferation following inhibition of JAG1 signaling by a neutralizing antibody. Our data showed that short-term blockade of JAG1 for 48 h was not associated with inhibition of cellular growth for ATL55T cells (Fig. 6c). In agreement with these findings, propidium iodide staining followed by FACS analysis demonstrated no significant cell death in ATL55T or ATL43T cells following transient inhibition of JAG1 (Fig. 6d). It is possible that JAG1 was not sufficiently inhibited or that alternative pathways are used. It is also possible that inhibition for longer periods are required to significantly halt ATL cell proliferation and survival. Additional studies may be warranted to answer these questions. Since JAG1 overexpression has been implicated in metastasis, we then investigated the role of JAG1 in ATL tumor cell migration by wound assays in vitro [25]. Results presented in Fig. 6e demonstrate a reduction in wound healing 48 h after inhibition of JAG1, suggesting that in ATL cells, high JAG1 expression may contribute to tumor cell migration in vitro.

## Discussion

In the present study, we investigated the molecular mechanisms that lead to constitutive Notch activation in HTLV-I-transformed and ATL cells. Among the Notch ligands, JAG1 was found to be significantly overexpressed both in virus-transformed cell lines and PBMCs isolated from acute ATL patients. Other Notch receptors, including JAG2, DLL1, and DLL4, were not significantly increased across all cells tested. JAG1 induction has been reported to affect both tumor cells and multiple components of the neoplastic microenvironment, including the vasculature and immune cells [26, 27]. Interestingly, several pieces of evidence demonstrate that JAG1 plays a role in some hematopoietic malignancies. For instance, in multiple myelomas, JAG1 is highly expressed and induces Notch activation, which in turn drives myeloma cell proliferation [28]. JAG1 also induces Notch over-activation in B cell chronic lymphocytic leukemia, and JAG1 stimulation in ex vivo cultures protects from spontaneous apoptosis [29, 30]. This demonstrates that JAG1 is important in sustaining the survival of cancer cells. It has also been reported that JAG1 overexpression by bystander and adjacent tumor cells leads to Notch1 activation and promotes cell growth in Hodgkin’s and anaplastic large cell lymphoma [31], suggesting that high expression of JAG1 might have a role in the activation of Notch1 in HTLV-1-induced leukemia.

Our studies demonstrated that the Tax viral protein stimulates JAG1 gene expression in part through Tax-mediated NF-κB activation and was associated with increased JAG1 cell surface expression. In contrast, the viral gene HBZ had no significant effects on JAG1 expression. We then showed that the microRNA, miR-124a, significantly inhibited the expression of JAG1 in ATL-derived cell lines. The underlying mechanism was identified as miR-124a-mediated direct targeting of JAG1 mRNA as well as miR-124a-targeting STAT3 and NFATc1, two transcriptional factors controlling JAG1 gene expression. Our previous study described decreased expression of miR-124a in an HTLV-I context [16], suggesting that the absence of a negative regulator might contribute to JAG1 overexpression both in cell lines and ATL patients even in the absence of Tax expression. Consistent with this notion, the expression of STAT3 and NFATc1 were directly correlated to that of JAG1 in primary ATL patients, and pharmacological inhibition of either STAT3 or NFATc1 was associated with decreased JAG1 expression in ATL cell lines.

Activation of the Notch signaling pathway is particularly relevant in HTLV-1-infected cells because its prolonged pharmacological inhibition significantly reduces tumor size in an engrafted ATL mouse model [13]. High expression of JAG1 has been associated with increased migration and invasion of tumor cells and metastasis and poor prognosis in non-small cell lung cancer (NSCLC) [25]. JAG1 is also highly expressed in medulloblastoma and colorectal cancer, and JAG1 causes poorer overall survival in breast cancer [32–34]. Studies have demonstrated that JAG1 signaling in cancer cells can activate downstream pathways such as AP-1 (activator protein 1), MAPK (mitogen-activated protein kinases), EGFR (epidermal growth factor receptor), and NF-κB [27, 35–37]. Along these lines, AP-1, MAPK, and NF-κB have also been shown to be activated in ATL cells. Whether JAG1 overexpression is involved in these processes warrants additional investigation. We also found that inhibition of JAG1 signaling by using a neutralizing antibody or shRNA does not affect the short-term proliferation or survival of ATL cells. It is possible that JAG1 inhibition is not sufficient to completely abrogate Notch1 activation. This notion is supported by the fact that blocking JAG1 reduced expression of Notch1 downstream targets (Hes-1, Hey-1, and VEGF) by 50%. However, our data suggest that inhibition of JAG1 even transiently is sufficient to significantly affect ATL tumor cell migration, which may be a function of JAG1 independent of Notch signaling and warrants additional studies.

## Conclusions

Our study demonstrates a significant overexpression of the Notch ligand JAG1 in ATL cells versus normal PBMCs. This overexpression was linked to viral Tax, miR-124a, STAT3, and NFATc1. JAG1 overexpression was associated with Notch1 signaling in ATL cells. Our data further suggests JAG1 as a possible candidate for the development of immunotherapy against ATL cells.
